# Potential Role of Circulating microRNA-21 for Hepatocellular Carcinoma Diagnosis: A Meta-Analysis

**DOI:** 10.1371/journal.pone.0130677

**Published:** 2015-06-26

**Authors:** Qibin Liao, Peiyu Han, Yue Huang, Zhitong Wu, Qing Chen, Shanshan Li, Jufeng Ye, Xianbo Wu

**Affiliations:** 1 School of Public Health and Tropical Medicine, Southern Medical University, Guangzhou, Guangdong, China; 2 Department of Epidemiology, Guangdong Provincial Key Laboratory of Tropical Disease Research, School of Public Health and Tropical Medicine, Southern Medical University, Guangzhou, Guangdong, China; 3 Experimental Teaching Center of Preventive Medicine, School of Public Health and Tropical Medicine, Southern Medical University, Guangzhou, Guangdong, China; Yonsei University College of Medicine, REPUBLIC OF KOREA

## Abstract

**Background:**

Circulating microRNA-21 (miR-21) is known to be aberrantly expressed in hepatocellular carcinoma (HCC) patients, and this implies that microRNA-21 is a promising and novel indicator of HCC. However, a systematic evaluation of the performance of microRNA-21 as a diagnostic marker for HCC has yet to be conducted. Therefore, the test performance of circulating miR-21 for HCC was assessed in this study.

**Methods:**

Three common international databases and a Chinese electronic database were used to search for literature on the diagnostic accuracy of microRNA-21 for HCC. The pooled results included the sensitivity and specificity of microRNA-21 for HCC detection and were analyzed with a random effect model. The area under summary receiver operating characteristic curve (AUC) was used to estimate overall test performance.

**Results:**

A total of 339 HCC patients and 338 controls without HCC from four published studies were eligible for the meta-analysis and included in our study. The test performance of circulating miR-21 in HCC detection was assessed with the summary estimates of sensitivity and specificity, which were 81.2% (95% CI: 70.8% to 88.4%) and 84.8% (95% CI: 75.1% to 91.2%), respectively. The value of AUC was 0.90 (95% CI: 0.87 to 0.92). Significant inter-study heterogeneity was detected by our analysis, and sub-group analyses suggested that the type of control group was probably a source of heterogeneity.

**Conclusions:**

Our current findings suggested that circulating miR-21 can serve as a potential co-biomarker for early-stage HCC diagnosis. Thorough large-scale studies are needed to confirm the generalizability of our findings.

## Introduction

Primary liver cancer is the fifth commonest malignancy and its mortality ranks second among all cancer-related deaths worldwide [1]. In 2012, the high incidence of liver cancer involves an estimated 782000 new cases and 746000 deaths globally [2]. Approximately 85 percent of primary liver cancers are hepatocellular carcinoma (HCC), which is the most commonly diagnosed histologic type [3]. The five-year survival rate of early-stage HCC patients is as high as 75% [4], whereas the 1-year survival rate for patients diagnosed with widespread cancer is less than 10% [5]. Therefore, surveillance and early diagnosis are of utmost importance. Currently, HCC surveillance relies on imaging and serological examinations. Ultrasonography(US) is the most commonly used imaging test for surveillance, which has a high specificity above 90%, however, it is less effective for the early detection of HCC due to the low sensitivity(63%)[6]. Owing to radiation exposure and high cost, computed tomography (CT)or magnetic resonance imaging (MRI) can not be recommended for routine HCC surveillance [7–8]. The usefulness of serological markers is limited. The most widely used tumor marker, serum alpha-fetoprotein (AFP), has contributed to a reduction in HCC mortality. However, increased AFP concentrations have been observed in Chronic Hepatitis B and/or C Patients without HCC [9]. Serum AFP level can be elevated in other types of non-liver cancer, including gastric cancer and cholangiocarcinoma [10–11]. Thus, AFP is not a specific marker for HCC diagnosis.

There are other common biomarkers of HCC, such as des-γ-carboxy prothrombin (DCP), glycosylated AFP-L3 fraction of total AFP and glypican 3 (GPC-3). Unfortunately, none have been found be specific of early-stage HCC [12]. Considering the above, minimally invasive and cost-effective biomarkers are therefore critically needed to screen and diagnose HCC.

MicroRNAs (miRNAs) are small (~22 nt) non-coding RNAs that have vital functions in various cellular biological processes, including cell proliferation, differentiation, and apoptosis [13]. It has been reported that aberrant expressions of miRNAs were in human cancers with differential regulation (“up” or “down”) observed in neoplastic compared with normal cells [14]. Several research groups reported that circulating miRNAs are stably detected and may be considered as a potential novel tool for human cancer detection [14–16].

MiRNA-21(miR-21) is one of the first oncogenic miRNAs with upregulation detected in many types of human cancer [17–19]. In recent years, the diagnostic value of circulating miR-21 in various human cancers has been studied intensely [20–22]. Several studies reported the significance of circulating miR-21 in HCC with inconsistent results [23–26]. For example, Xu et al reported miR-21 has a low specificity of 73.5%, however, a higher specificity (92.0%) was demonstrated by Tomimaru et al [23–24]. Therefore, we aimed to evaluate the potential role of circulating miR-21 in HCC diagnosis, which has not been previously investigated to the best of our knowledge.

## Methods

### Search strategy

A literature search (up to November 30, 2014) was performed on three international electronic databases (PubMed, Embase, and Web of Science) and a Chinese database (Chinese National Knowledge Infrastructure, CNKI). No limitations were imposed on language. The following two sets of keywords were used to retrieve relevant studies: (a) keywords for the primary disease: “hepatocellular cancer” or “hepatocellular tumor” or “hepatocellular carcinoma” or “hepatocellular neoplasm” or “liver cancer” or “liver tumor” or “liver carcinoma” or “liver neoplasm” or “HCC” and (b) keywords for the diagnostic biomarker: “microRNA-21” or “miRNA-21” or “miR-21” or “hsa-miR-21.” Our literature searches were limited to human studies. To validate the qualified studies, we also manually searched the references of review papers and other relevant studies.

### Selection criteria of the studies

All publications were carefully assessed by two authors (ZTW and QBL). The third reviewer (JFY) helped resolve discrepancies through discussion with the first two authors. The criteria for inclusion were as follows: (a) histopathological examination was used as the gold standard for HCC diagnosis; (b) the diagnostic studies employed circulating miR-21 to diagnose HCC; (c) the studies had sufficient data to calculate the number of true positive (TP), false positive (FP), false negative (FN), and true negative (TN) results; (d) serum and/or plasma samples were collected before surgery and chemotherapy; (e) each individual study involved two treatment groups, namely, the HCC and control groups.

In addition, the following exclusion criteria were used: (a) non-original papers, such as conference abstracts, letters, and reviews; (b) duplicate studies; and (c) studies without qualified data or with 20 patients or less.

### Data extraction and quality assessment

Data were independently extracted by two authors (ZTW and QBL) from the included studies. The following data were obtained: (a) study characteristics (first author, country, publication year, specimen collected, and assay method); (b) characteristics of the subjects (mean/median age, sample size, gender and etiologial factor); (c) sensitivity, specificity.

The quality assessment of each included study was performed with Quality Assessment of Diagnostic Accuracy Studies (QUADAS) [27]. This tool is considered reliable for the quality assessment of test accuracy studies.

### Statistical analysis

Meta-DiSc 1.4 and Stata 12.0 were used to perform all statistical analyses [28]. *P* < 0.05 denoted statistical significance. The between-study heterogeneity was evaluated with the *I* ^2^ index and χ^2^ test, such that a value of *I* ^2^ > 50% and/or a *p* < 0.05 indicated heterogeneity [29]. A random effects model was employed to pool statistical indices, including sensitivity, specificity, likelihood ratio (LR; positive LR/negative LR), and diagnostic odds ratio (DOR), when heterogeneity was present. In addition, meta-regression was further applied to explore possible sources of heterogeneity. The threshold effect was investigated based on the Spearman correlation coefficient. The overall diagnostic performance of the included studies was evaluated from a summary receiver operating characteristic curve (SROC). Finally, Deeks’ funnel plot analysis was performed to explore the potential publication bias [30].

## Results

### Study selection and quality assessment

**Fig 1:**
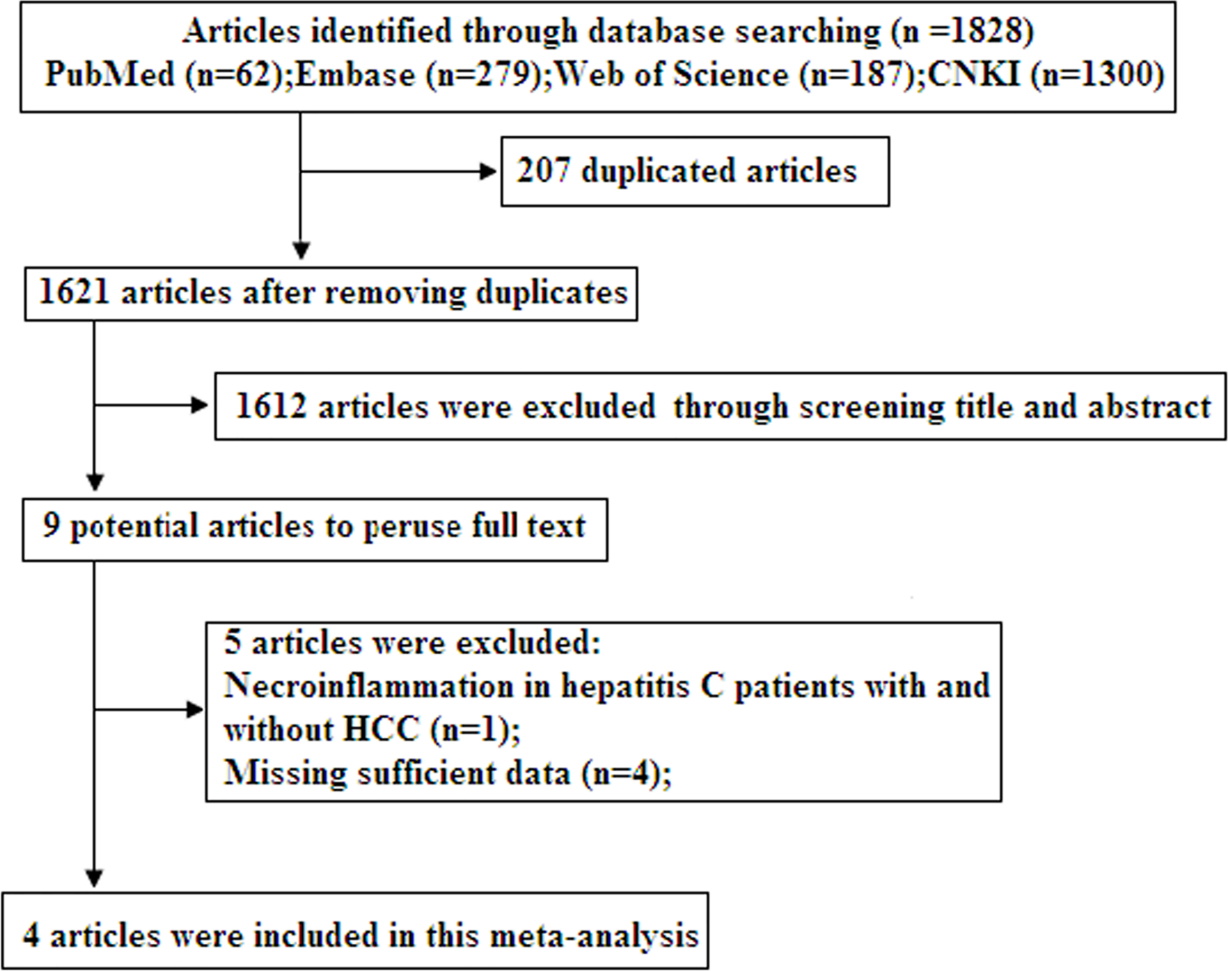
Flow diagram of study selection.

The literature search identified 1,828 relevant articles, 207 of which were excluded as duplicated publications. After a preliminary review of titles and abstracts, a total of 1,612 articles (reviews, case reports, letters, and studies not solely focused on HCC and/or not specifically pertaining to miR-21) were excluded for various reasons. The full-text articles of the nine remaining publications were obtained, and five studies were excluded for insufficient data. Finally, four publications were selected for our meta-analysis [23–26]. The flow diagram of study selection is summarized in Fig 1.

The four selected publications described six eligible studies and were published from 2011 to 2013; the data involved 339 HCC patients and 338 healthy or chronic hepatitis controls. Three of the articles were published in English, whereas the study by Qin et al. was in Chinese [26]. Histopathological examination was considered the gold standard for HCC diagnosis, and real-time quantitative PCR (RT-PCR) was used to test for miRNA-21. The detailed characteristics of these studies are listed in Table 1. QUADAS results showed generally high scores for all of the included papers (S1 Table.).

**Table 1:** Main characteristics of the studies included in the meta-analysis.

Study	Country	Patients (B+; B-C-/B+C-/ B-C+/B+C+)^†^	Controls (B+; B-C-/ B+C-/ B-C+/B+C+)	Age	Male (%)	Se%	Sp%
Xu *et al*.2011	China	101 (76; NR)	89^a^(0)	50	77	84	73.5
Tomimaru *et al*. (1) 2012	Japan	126(28; 14/25/84/3)	50^a^(0)	63	79	87.3	92.0
Tomimaru *et al*. (2) 2012	Japan	126(28;14/25/84/3)	30^b^(4; 0/4/26/0)	63	79	61.1	83.3
Liu *et al*. 2012	China	57 (57; NR)	59^c^(29; NR)	60	71	89.47	71.19
Qin *et al*. (1) 2013	China	55(21; 9/15/25/6)	50^a^(0)	64	55	89	95
Qin *et al*. (2) 2013	China	55(21; 9/15/25/6)	60^b^(25; 0/25/35/0)	64	55	65	85

^†^ B−, B+, C−, and C+ represent negative HBs-Ag, positive HBs-Ag, negative anti-HCV Ab, and positive anti-HCV Ab, respectively.

^a^, healthy control group;

^b^, control group with chronic hepatitis;

^c^, non-cancerous control group (healthy and chronic hepatitis B carriers).

Age, mean or median age of HCC patients; Male, men with HCC; Se, sensitivity; Sp, specificity;NR = not report.

### Overall analysis

**Fig 2:**
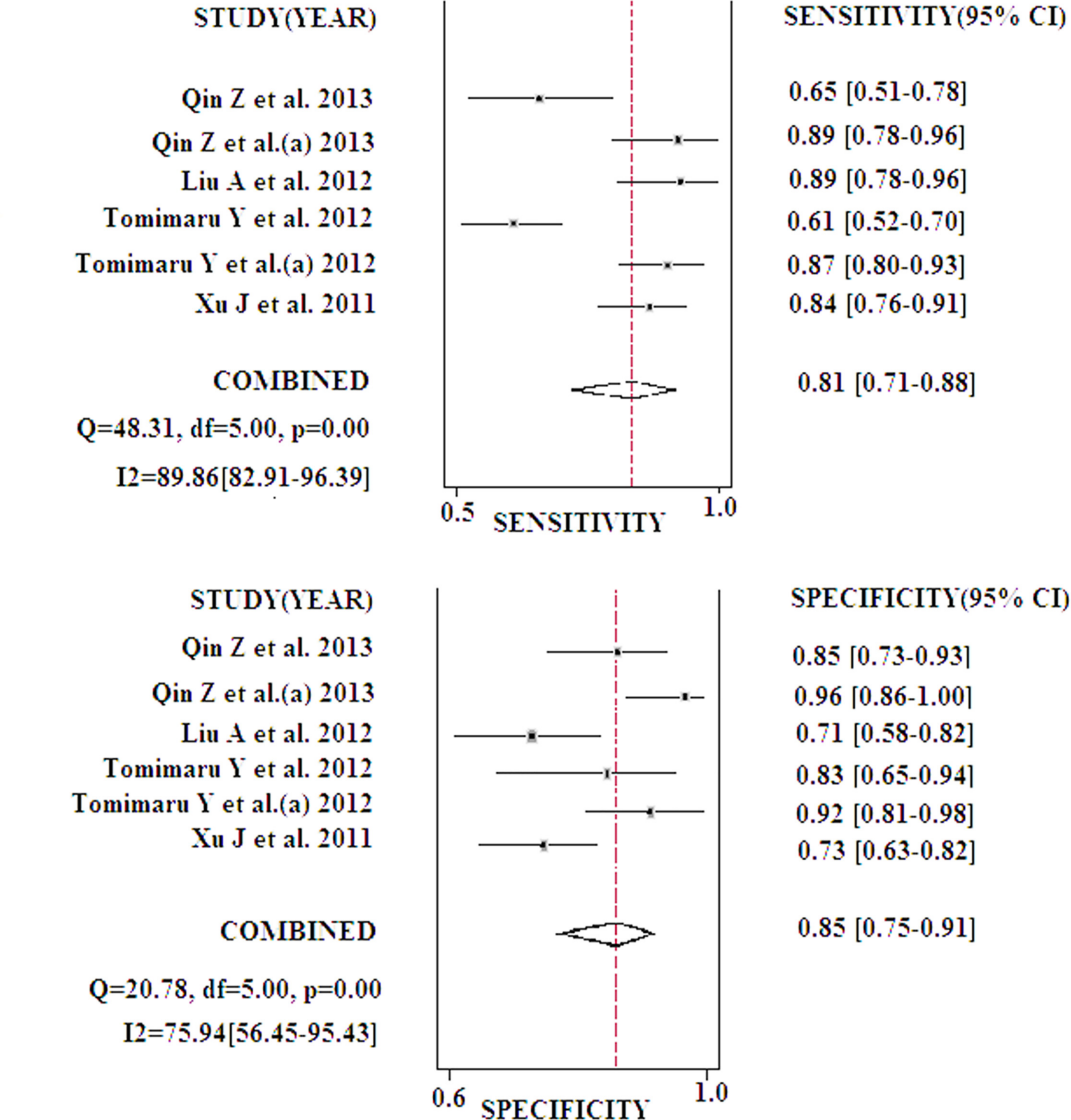
Forest plot of sensitivities and specificities from the test accuracy of circulating miR-21 in the diagnosis of HCC.

**Fig 3:**
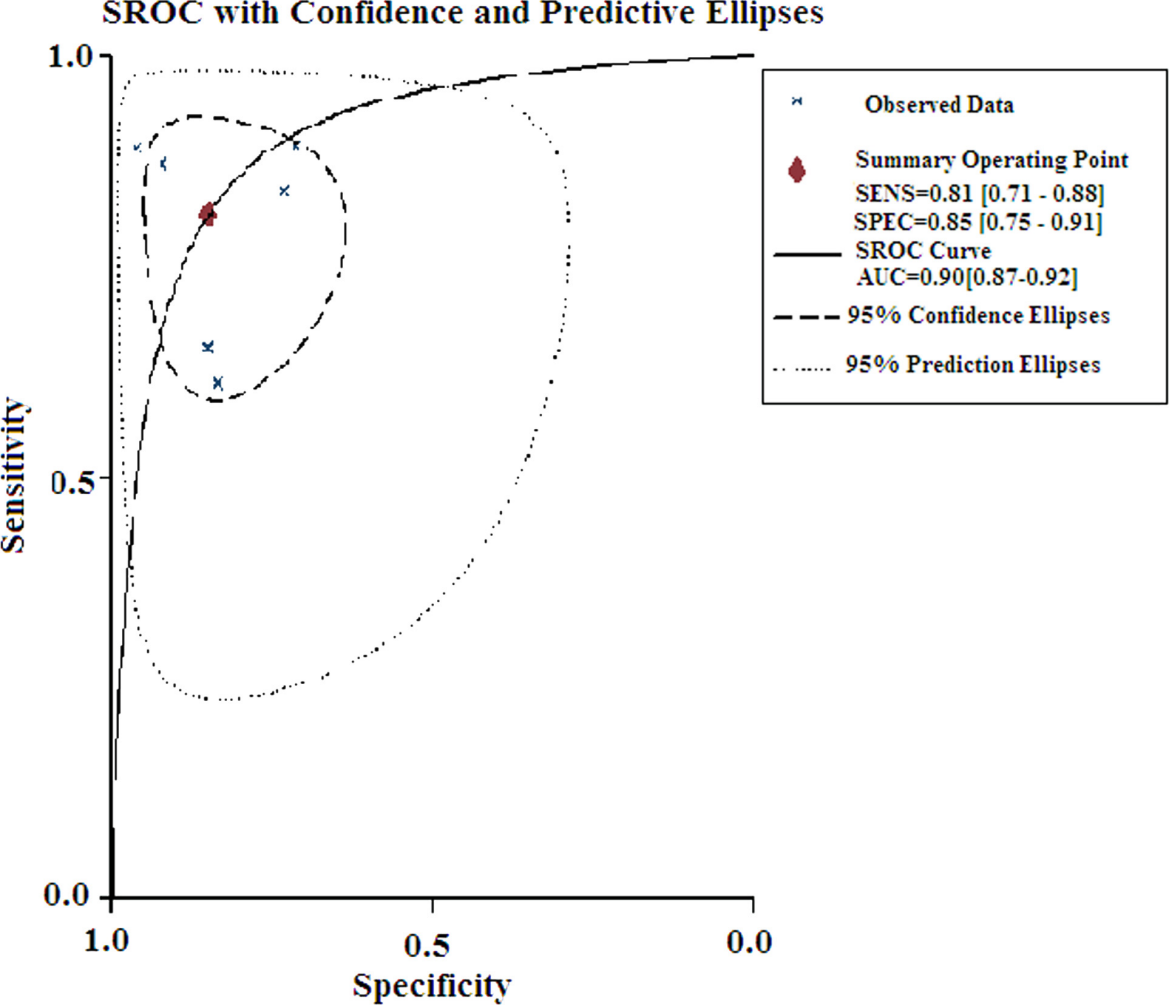
Summary receiver operating characteristic curves for miR-21 in the diagnosis of HCC.

Heterogeneity was present in our meta-analysis, as confirmed by the results (χ^2^ = 11.255, *p* = 0.002; *I* ^2^ = 82.23%). Therefore, a random effects model was selected. The pooled sensitivity and specificity of circulating miR-21 for HCC detection is summarized in Fig 2. The summary sensitivity and specificity for the pooled data were 81.2% (70.8% to 88.4%) and 84.8% (75.1% to 91.2%), respectively. The AUC was 0.90 (0.87 to 0.92; Fig 3), and the DOR was 24.038 (10.02 to 57.67). The results corresponded to a PLR of 5.34 (3.10 to 9.18) and an NLR of 0.22 (0.14 to 0.36). Significant heterogeneity was noted in the pooled sensitivity (*I* ^2^ = 89.65%) and specificity (*I* ^2^ = 75.94%) results.

### Threshold effect and heterogeneity

Threshold effect occurs when differences in sensitivities and specificities arise; this effect can be assessed with the Spearman correlation coefficient [28]. A value of 0.100 (*p* = 0.873; *p* > 0.05) indicated the absence of the threshold effect in our meta-analysis.

With the exception of variances originating from the threshold effect, heterogeneity can be caused by other factors, such as different clinical or socio-demographic characteristics and differences in the study design [31]. Meta-regression analysis was employed to investigate the possible sources of heterogeneity generated by the non-threshold effect. We initially considered two factors that may contribute to heterogeneity, namely, age and gender. A meta-regression analysis was performed to confirm whether age and gender were sources of heterogeneity. Insignificant heterogeneity was noted among studies in terms of age (coefficient = 0.066, *p* = 0.78) and gender (coefficient = -2.148, *p* = 7.86). Therefore, age and gender were probably not the sources of heterogeneity, and others factors might have caused the observed heterogeneity.

### Sub-group analyses

The results from 282 HCC patients and 189 healthy individuals in three studies [23–24,26] were pooled. The summary sensitivity and specificity of circulating miR-21 for discriminating HCC from healthy individuals were 86.5% (82.0% to 90.3%) and 84.1% (78.1% to 89.0%), respectively. The PLR, NLR, DOR, and AUC were 8.31 (2.02 to 34.10), 0.16 (0.11 to 0.23), 53.66 (11.0 to 261.7), and 0.93, respectively. Additionally, we found that absence of heterogeneity in the sensitivity values (*I* ^2^ = 0.0%, *p* = 0.651), a result suggesting that the type of control group may be the source of heterogeneity. However, the pooled sensitivity and specificity of circulating miR-21 for discriminating HCC from chronic hepatitis were 62.4% (54.9% to 69.5%) and 84.4% (75.3% to 91.2%) based on the results for 181 HCC and 90 individuals with chronic hepatitis [24,26], respectively.

### Publication bias

**Fig 4:**
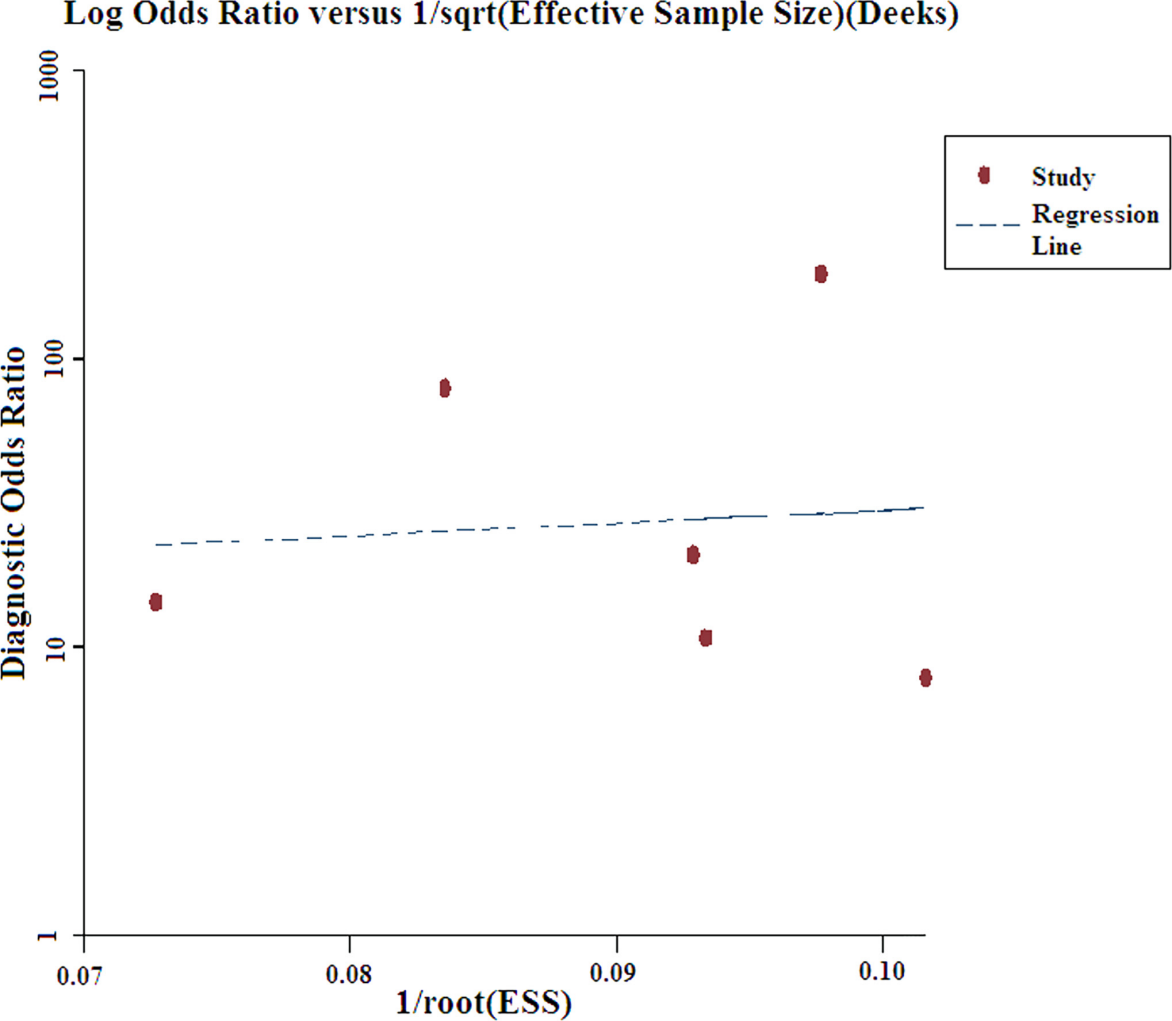
Funnel plot for the assessment of potential publication bias in miR-21 assays.

To explore the potential publication bias among the included studies, Deeks’ funnel plots were designed in our meta-analysis. The obtained *p*-value of 0.856 suggested the absence of publication bias (Fig 4).

## Discussion

Increasing evidence has demonstrated that circulating miR-21 is involved in cancer diagnosis and prognosis. The diagnostic value of miR-21 for gastric cancer and lung cancer was evaluated in previous reports [21–22]. However, a meta-analysis of the potential role of blood-based miR-21 for the diagnosis of HCC has not been reported to date. The results of our meta-analysis indicated an overall moderate test performance of circulating miR-21 for the diagnosis of HCC. The pooled sensitivity of plasma/serum miRNA-21 (81.2%) showed superiority in diagnosis compared with that of AFP, with an overall sensitivity less than 60% [24]. Therefore, circulating miR-21 was a potentially biomarker for HCC diagnosis. An AUC of 0.93 showed that circulating miR-21 may be a promising marker to discriminate HCC patients from health individuals, with a summary sensitivity of 86.5% and specificity of 84.1%. Whether circulating miR-21 could be used to differentiate HCC from chronic hepatitis should be investigated because 80%–90% of HCC patients have an established background of chronic hepatitis B and/or C and liver cirrhosis [31–32]. Our analysis of the combined studies by Tomimaru Y et al. and Qin Z et al. suggested that the pooled sensitivity and specificity of miR-21 for differentiating HCC from volunteers with chronic hepatitis were 62.4% and 84.4%, respectively.

Significant heterogeneity was noted in this meta-analysis (*I* ^2^ = 82.23%), and the value of the Spearman correlation coefficient was 0.100 (*p* > 0.05), which indicated the lack of heterogeneity caused by the threshold effect. Meta-regression analyses suggested that age and gender may not be the potential sources of heterogeneity among the studies, whereas the sub-group analyses suggested that the type of control group was probably a source of heterogeneity. Although the included studies have all been published, these studies originated from a small group of authors. Therefore, the reliability of the conclusions of our meta-analysis may be affected by publication bias. However, the funnel plot showed the absence of publication bias.

As a potential diagnostic biomarker for HCC, circulating miR-21 possesses several unique advantages. First, serum or plasma microRNA is characterized by minimal invasion and convenience compared with histopathological examination. Second, serum microRNA expression levels are stable and reproducible [16]. Third, plasma miR-21 level cannot be influenced by both cirrhosis and viral status. Fourth, Significant overexpression of plasma miRNA-21 was observed even in patients with early-stage HCC [24]. On the other hand, AFP level of 400 ng/ml is considered as an indicator of HCC in general, which does not occur at an early HCC stage. As a result, about one-third of all HCC case with small lesions(<3cm) were not diagnosed in the early tumor stage [33–34]. Therefore, circulating miR-21 may serve as a novel co-biomarker to AFP to improve the diagnostic accuracy of early-stage HCC.

Notably, certain limitations were present in our study. First, the overexpression of circulating miRNA-21 was previously reported for other human tumors, such as gastric and lung cancer [35–36]. This phenomenon indicates that the increased expression of miR-21 may not be specifically associated with HCC itself, which is common in the progression of various cancers. Therefore, miRNA-21 alone may not be a specific indicator for the diagnosis of HCC in routine clinical practice, but this marker will improve test performance when it is used in combination with other biomarkers. Tomimaru et al. suggested that the combination of plasma miR-21 and AFP enhanced the performance of AFP in discriminating HCC from healthy volunteers (AUC = 0.971) and patients with chronic hepatitis (AUC = 0.823) [24]. Second, the significance of circulating miR-21 in HCC has not been reported until 2011 [23]. Circulating miR-21 as a novel biomarker for HCC just recently attracts researchers’ attention, and still limited studies were performed on the topic. Therefore, only a relatively small study size can be obtained in the meta-analysis. Given the small number of cases from the limited number of studies in the present meta-analysis, we could not conduct meta-regression analyses of more factors to further explore heterogeneity. Third, HCC is an especially common cancer in China and Japan. Over 50 percent of the worldwide HCCs occur in Chinese registries alone, and high incidence of HCC is also be seen in Japan, with approximately 40 cases per 100,000 persons [1]. More researchers of these areas were thus engaged in studying HCC. Consequently, the available papers included in the meta-analysis are all published from China and Japan. Therefore, large-scale and multi-center studies are required to confirm our findings, especially the potential use of circulating miRNA-21 in discriminating HCC from chronic hepatitis and cirrhosis. Finally, the cost and technical issues involved in testing for miR-21 in clinical practice should be taken into consideration.

Overall, our study is the first meta-analysis to assess the potential role of circulating miR-21 for HCC. Our meta-analysis demonstrated that miRNA-21 presents moderate diagnostic performance and can serve as a potential co-biomarker for early-stage HCC diagnosis. However, future large-scale studies are important to validate the potential usefulness of miR-21.

## Supporting Information

S1 Checklist PRISMA checklist. (DOC)

S1 Table Quality Assessment of Diagnostic Accuracy Studies (QUADAS). (DOC)
