# Supplementary material for: Potential Role of Circulating microRNA-21 for Hepatocellular Carcinoma Diagnosis: A Meta-Analysis
Source: PLoS One. 2015 Jun 26;10(6):e0130677. doi: 10.1371/journal.pone.0130677 (PMC4483261; doi:10.1371/journal.pone.0130677)
Supplement: S1 Checklist — (DOC) [file pone.0130677.s001.doc]

| **Section/topic** | **#** | **Checklist item** | **Reported on page #** |
| --- | --- | --- | --- |
| **TITLE** | | |  |
| Title | 1 | A meta-analysis of diagnostic value of microRNA-21 for hepatocellular carcinoma | 1 |
| **ABSTRACT** | | |  |
| Structured summary | 2 | **Abstract**  ***Background*:** Previous studies have demonstrated that circulating microRNA-21 (miR-21) was aberrantly expressed in hepatocellular carcinoma (HCC) patients, indicating that microRNA-21 may be a promising and novel indicator to identify HCC. However, a systematic evaluation of the performance of microRNA-21 as a diagnostic marker for HCC has yet to be conducted. Therefore, the test performance of miR-21 for HCC was assessed in our study.  ***Methods*:** Three common national databases and a Chinese electronic database were used to search for the literature reporting the diagnostic accuracy of microRNA-21 for HCC. The pooled results, such as sensitivity and specificity of microRNA-21 for CRC detection, were calculated using a random effect model. Area under summary receiver operating characteristic curve (AUC) was employed to estimate the overall test performance.  ***Results*:** studies with 339 HCC patients and 338 controls without HCC were eligible and included in our study. The test performance of circulating miR-21 in HCC detection are as follows: summary estimates of sensitivity and specificity were 81.2% (95% CI: 70.8% to 88.4%) and 84.8% (95% CI: 75.1% to 91.2%). The value of AUC was 0.90 (95% CI: 0.87 to 0.92). Significant inter-study heterogeneity existed in our study, while age and sex may be not potential sources of heterogeneity.  ***Conclusions*:** Our current findings suggested that circulating miR-21 may be a promising marker for diagnosis of HCC. Further large-scale and multi-center studies are needed to confirm the generalizability of our findings. | 2 |
| **INTRODUCTION** | | |  |
| Rationale | 3 | Several research groups have now published their studies about the test performance of circulating miR-21 for diagnosis of HCC, and inconsistent results raise concerns on the diagnostic value of circulating miR-21 for detection of HCC | 2-3 |
| Objectives | 4 | In light of these inconsistent studies, we aimed to perform a comprehensive meta-analysis to confirm whether blood-based miRNA-21 could be considered as a promising biomarker for HCC diagnosis. | 2-3 |
| **METHODS** | | |  |
| Protocol and registration | 5 | There is no review protocol existed. |  |
| Eligibility criteria | 6 | The criteria for inclusion were as follows: (1) histopathological examination is the golden standard of CRC diagnosis; (2) diagnostic studies used circulating miR-21 to diagnose human CRC; (3) studies should contain sufficient data to calculate the numbers of true positive (TP), false positive (FP), false negative (FN), and true negative (TN); (4) serum and/or plasma samples were collected before surgery and chemotherapy; (5) individual study containing two comparator groups, namely, CRC group and control group. | 3-4 |
| Information sources | 7 | PubMed, Embase, Web of Science and Chinese National Knowledge Infrastructure (CNKI) | 3-4 |
| Search | 8 | A literature search (up to November 30th, 2014) was performed via the following three national electronic databases (PubMed, EMBASE and Web of Science) and a Chinese database (CNKI). No limitations were imposed on language. Two sets of key words used to retrieve relevant studies were as follows: (a) the primary disease: “hepatocellular cancer” or “hepatocellular tumor” or “hepatocellular carcinoma” or “hepatocellular neoplasm” or “liver cancer” or “liver tumor” or “liver carcinoma” or “liver neoplasm” or HCC; (b) the diagnostic biomarker: microRNA-21 or miRNA-21 or miR-21 or hsa-miR-21. Our search was limited to human studies. To validate the qualified studies, we also hand-searched the references of the review papers. | 3-4 |
| Study selection | 9 | All publications were carefully assessed by two authors (ZTW and QBL). The third reviewer (JFY) helped to resolve discrepancies through discussing with the two authors. | 4 |
| Data collection process | 10 | Data extracted by two authors (ZTW and QBL) independently from the included studies were as follows: (1) study characteristics (author, publication country and year, specimen and assay method); (2) characteristics of subjects (mean/median age, sample size and sex); (3) sensitivity, specificity and AUC. | 4 |
| Data items | 11 | (1) study characteristics (author, publication country and year, specimen and assay method); (2) characteristics of subjects (mean/median age, sample size, sex); (3) sensitivity, specificity and AUC. | 4 |
| Risk of bias in individual studies | 12 | The potential publication bias of selected studies was assessed using the funnel plot. | 4 |
| Summary measures | 13 | The pooled sensitivity, specificity, likelihood ratio (PLR & NLR), diagnostic odd ratio (DOR) and their 95% confidence intervals (CIs) were calculated using a random effects model. Simultaneously, we generate the summary receiver operator characteristic (SROC) curve and calculate the area under the SROC curve (AUC). | 4 |
| Synthesis of results | 14 | Analyses were performed using Stata SE12.0 and Meta-Disc software, version 1.4. Pooled sensitivity and specificity, PLR, NLR, SROC and DOR were calculated. Consistency was done by Q-test and χ^2^ test. | 4 |

Page 1 of 2

| **Section/topic** | **#** | **Checklist item** | **Reported on page #** |
| --- | --- | --- | --- |
| Risk of bias across studies | 15 | publication bias, selective reporting within studies. | 4 |
| Additional analyses | 16 |  |  |
| **RESULTS** | | |  |
| Study selection | 17 | The flow diagram of study selection was summarized in Figure 1. A total of 4 article including 6 eligible studies met the inclusion criteria were included in this meta-analysis. | 5 |
| Study characteristics | 18 | (1) study characteristics (author, publication country and year, specimen and assay method); (2) characteristics of subjects (mean/median age, sample size, sex); (3) sensitivity, specificity and AUC. | 5 |
| Risk of bias within studies | 19 | Since publication bias is concerned for diagnostic meta-analysis, Deeks’ funnel plots were used to explore the potential publication bias among these included studies. The p value of 0.856 indicated that no publication bias existed in the present study. | 6 |
| Results of individual studies | 20 | None |  |
| Synthesis of results | 21 | Heterogeneity exists in this meta-analysis, as confirmed by the results (χ^2^ = 11.255, p=0.002; *I*^2^ = 82.23%). A random effect model was therefore selected. Figure 2 shows the pooled sensitivity and specificity of circulating miR-21 for HCC detection, respectively. The summary sensitivity and specificity for pooled data were 81.2% (70.8% to 88.4%) and 84.8% (75.1% to 91.2%), respectively. AUC was 0.90 (0.87 to 0.92) (Figure 3), and DOR was 24.038 (10.02 to 57.67). The results corresponded to a PLR of 5.34 (3.10 to 9.18) and a NLR of 0.22 (0.14 to 0.36). Significant heterogeneity was noted in the pooled sensitivity (*I*^2^ =89.65%) and specificity (*I*^2^=75.94%). | 5-6 |
| Risk of bias across studies | 22 | None |  |
| Additional analysis | 23 | None |  |
| **DISCUSSION** | | |  |
| Summary of evidence | 24 | Increasing evidence has demonstrated that circulating miR-21 is involved in cancer diagnosis and prognosis. Evaluation of the diagnostic value of miR-21 for gastric cancer and lung cancer previously been reported. However, meta-analysis concerning the diagnostic accuracy of blood-based miR-21 for diagnosis of hepatocellular carcinoma has not been reported to date. The results of our meta-analysis indicated an overall moderate test performance of circulating miR-21 for diagnosis of HCC. The pooled sensitivity of plasma/serum miRNA-21 (81.2%) showed superiority in comparison with AFP in diagnosis with an overall specificity less than 65%, which suggested that circulating miR-21 was potentially a substantially more suitable biomarker for the diagnosis of HCC. The AUC of 0.93 showed that circulating miR-21 may be promising marker for discriminating HCC patients from health individuals, with the summary sensitivity of 86.5% and specificity of 84.1%. It is also important to investigate whether circulating miR-21 could be used to differentiate HCC from chronic hepatitis due to the fact that 80%–90 % of HCC patients have an established background of chronic B and /or C hepatitis and liver cirrhosis. The analysis of studies of Tomimaru Y et al. and Qin Z et al. suggested that the pooled sensitivity and specificity of miR-21 for differentiating HCC from volunteers with chronic hepatitis were 62.4% and 84.4%, respectively. | 6 |
| Limitations | 25 | As a potential diagnostic biomarker for HCC, circulating miR-21 possesses two unique superiorities. On the one hand, serum or plasma microRNA is characterized by minimal invasion and convenience in comparison with histopathological examination. On the other hand, serum microRNAs expression levels are more stable and reproducible (28), therefore, circulating miR-21 may serve as a novel tool to differentiate HCC patients from healthy individuals and/or chronic liver diseases. Notably, certain limitations emerged in our study. First, over-expression of circulating miRNA-21 has previously been reported in other human tumors, such as gastric cancer and lung cancer. This phenomenon indicates that the increased expression of miR-21 may not be specifically associated with HCC itself, commonly existing in the progression of numerous cancers. Therefore, single miRNA-21 may not serve as a specific indicator for the diagnosis of HCC in routine clinical practice, it will improve test performance with the combination of other biomarkers. Tomimaru Y et al. suggested that the combination of plasma miR-21 and AFP enhanced the performance of AFP in discriminating HCC from healthy volunteers (AUC=0.971) and patients with chronic hepatitis (AUC=0.823). Second, owing to small number of cases in the limited studies included in this meta-analysis, we could not conduct meta-regression analyses of more factors to further explore heterogeneity, thus, large-scale and multi-center studies are required to confirm our findings, especially the potentiality of niR-21 used for discriminating HCC from chronic hepatitis and cirrhosis. Last but no least, we should take the cost and technical issues involving in testing miR-21 into consideration in clinical practices. | 7 |
| Conclusions | 26 | Overall, our study is the first meta-analysis to assess the diagnostic value of circulating miRNA-21 for HCC, including the performance of miR-21 for discriminating healthy individuals and/or patients with chronic hepatitis.. Our meta-analysis demonstrates that miRNA-21 presents a moderate diagnostic performance, which is a minimally invasive biomarker for HCC detection. However, large-scale studies are needed to validate the potential usefulness of miR-21 in the future. | 7 |
| **FUNDING** | | |  |
| Funding | 27 | This study was financially supported by the Presidential Foundation of the School of Public Health and Tropical Medicine, Southern Medical University, China (No.GW201423). | 8 |

*From:*  Moher D, Liberati A, Tetzlaff J, Altman DG, The PRISMA Group (2009). Preferred Reporting Items for Systematic Reviews and Meta-Analyses: The PRISMA Statement. PLoS Med 6(6): e1000097. doi:10.1371/journal.pmed1000097

For more information, visit: **www.prisma-statement.org**.

Page 2 of 2
