# Supplementary material for: Potential Role of Circulating microRNA-21 for Hepatocellular Carcinoma Diagnosis: A Meta-Analysis
Source: PLoS One. 2015 Jun 26;10(6):e0130677. doi: 10.1371/journal.pone.0130677 (PMC4483261; doi:10.1371/journal.pone.0130677)
Supplement: S1 Table — (DOC) [file pone.0130677.s002.doc]

| Item No. | Description | Xu J  et al[15] | Tomimaru Y  et al[16] | Liu A  et al[17] | Qin Z  et al[18] |
| --- | --- | --- | --- | --- | --- |
| 1 | Representative spectrum | N | N | N | N |
| 2 | The clear selection criteria | Y | Y | Y | Y |
| 3 | Acceptable reference standard | Y | Y | Y | Y |
| 4 | Acceptable delay between tests | Y | Y | Y | Y |
| 5 | Partial verification | Y | Y | Y | Y |
| 6 | The same reference test regardless of the index test in detail | Y | Y | Y | Y |
| 7 | Incorporation bias | Y | Y | Y | Y |
| 8 | The execution of the index test in detail | Y | Y | Y | Y |
| 9 | The reference standard in detail | U | U | U | U |
| 10 | The index test results was blinded to the reference test results | U | U | U | U |
| 11 | The reference standard was blinded to the index test results | Y | Y | Y | Y |
| 12 | The availability of clinical data that would be available in clinical practice when using the index test | Y | Y | Y | Y |
| 13 | Reporting of uninterpretable results | U | U | U | U |
| 14 | Explanation of withdrawals from the study | Y | Y | Y | Y |

Table S1 The quality assessment of diagnostic accuracy studies.
